# Ethanol Consumption by Wistar Rat Dams Affects Selenium Bioavailability and Antioxidant Balance in Their Progeny

**DOI:** 10.3390/ijerph6082139

**Published:** 2009-07-30

**Authors:** María Luisa Ojeda, Beatriz Vázquez, Fátima Nogales, María Luisa Murillo, Olimpia Carreras

**Affiliations:** Department of Physiology and Zoology, Faculty of Pharmacy, Seville University /c/ Tramontana s/n. C.P. 41012. Seville, Spain; E-Mails: ojedamuri11@us.es (M.L.O.); beatriz_vazqueza@hotmail.com (B.V.); fnogales@us.es (F.N.); taravillo@us.es (M.L.M.)

**Keywords:** Wistar rats, selenium bioavailability, ethanol, gestation, lactation, glutathione peroxidase

## Abstract

Ethanol consumption affects maternal nutrition, the mothers’ antioxidant balance and the future health of their progeny. Selenium (Se) is a trace element cofactor of the enzyme glutathione peroxidase (GPx). We will study the effect of ethanol on Se bioavailability in dams and in their progeny. We have used three experimental groups of dams: control, chronic ethanol and pair-fed; and three groups of pups. Se levels were measured by graphite-furnace atomic absorption spectrometry. Serum and hepatic GPx activity was determined by spectrometry. We have concluded that ethanol decreased Se retention in dams, affecting their tissue Se deposits and those of their offspring, while also compromising their progeny’s weight and oxidation balance. These effects of ethanol are caused by a reduction in Se intake and a direct alcohol-generated oxidation action.

## 1. Introduction

It is known that ethanol consumption compromises oxidative stress and nutritional status [1]. Patients with chronic alcohol abuse present malnourishment, either because of reduced intake of essential nutrients, or because alcohol precludes an appropriate absorption of essential elements. Furthermore, metabolic ethanol pathways themselves generate intermediate toxic products (acetaldehyde and free radicals) that interfere with the normal metabolism of essential elements, leading to cellular damage through oxidation mechanisms and secondary oxidative stress [2] inflammation.

Only recently have studies begun to investigate the influence of alcohol on nutrition and endocrine function during pregnancy. Alcohol-induced changes in maternal endocrine function disrupt maternal-foetal hormonal interactions and affect the female’s ability to maintain a successful pregnancy, thus indirectly affecting the foetus [3]. Moreover foetal ethanol exposure specifically increases ethanol avidity, so ethanol abuse has broad implications in the relationship between maternal consumption, child development, and postnatal vulnerability [4].

As all essential trace elements required by offspring development are transferred from the dam via either the placenta or milk, if an insufficient milk intake occurs or if there is a disturbance in pups’ intestinal permeability (both effects are provoked by ethanol exposure in pups [5]), the low maternal concentration of immunoglobulines and antioxidant nutrients may be transferred [6].

One of the essential trace elements which could be compromised by ethanol consumption is Selenium. Se intake is important to health due to its anti-inflammatory, chemopreventive and antioxidant activity via different selenoproteins [7]. The most studied of these is the antioxidant enzyme GPx. There is increasing evidence that Se is vital for foetal and neonatal development. This is demonstrated by the embryonically lethal consequences of the disruption of the gene coding for the Sec tRNA^[Ser]Sec^, suggesting an essential function for one or more selenoproteins in development [8]. Moreover neonates have Se deposits but they need Se via milk for an infant’s optimum Se status [9]. We have recently observed that pups exposed to ethanol during gestation and lactation have their hepatic antioxidant activity altered, provoking a decrease in Se and GPx activity, and an increase in carbonyl groups in protein. Administering Se with alcohol to their mothers balances the hepatic oxidation process, suggesting that Se could be effective in neutralising the oxidative damage of ethanol consumption during gestation and lactation in pups [10].

The objective of this study was to examine the bioavailability of Se in both dams exposed to ethanol during gestation and lactation and in their progeny. In this context we will evaluate the activity of the antioxidant enzyme GPx in pups’ serum and liver, and the relationship with maternal nutrition.

## 2. Results and Discussion

### 2.1. Dams' Weight Gain and Se Homeostasis

As we observe in Table 1, alcohol and pair-fed dams showed a significant decrease in selenium intake during gestation and the lactation period (p < 0.05), this was reflected in their body weights (p < 0.01) and in the amount of Se excreted in faeces (p < 0.001). However PD eliminated less Se via urine than the rest of the groups (p < 0.001) but more via claws and hair (p < 0.001), and ethanol dams excreted less Se via hair than control ones (p < 0.001). With respect to control dams, alcohol consumption does not affect the apparent Se absorption rate, but it significantly decreases the apparent Se balance (p < 0.001), although serum Se levels were similar. However, with respect to CD, PD had a higher apparent Se absorption rate (p < 0.05) but a lower apparent Se balance and serum Se levels (p < 0.001).

Confirming our experimental treatments, ethanol and pair-fed dams consume less food and consequently less Se, and they also show a lower weight gain. Despite the fact that ethanol consumption appears not to change Se absorption, pair-fed dams have a higher apparent Se absorption than control ones. With respect to Se intestinal absorption, ethanol *per se* slightly alters Se absorption, this effect of alcohol on intestinal absorption also occurs with other nutrients [5,11]. Isocaloric dams eliminate less Se via urine than their ethanol counterparts. This fact, together with the changes in apparent absorption means that alcohol-exposed rats have the lowest apparent Se balance. This decrease affects their Se tissue deposits. Ethanol does not, however, affect serum Se levels, which decreased dramatically in pair-fed dams. This might occur because isocaloric rats eliminate much more Se by other excretion routes such as claws and hair [12]. Pair-fed dams have nearly twice the Se concentration in claws and hair than controls yet alcohol-consuming dams have three times less Se in hair than controls. We have no answer for the reason why pair-fed lactating dams and not ethanol-exposed ones excrete less Se via urine and lung (two of the greatest Se excretion routes [13]) and more via claws and hair. On the other hand, it is assumed that a high hair Se concentration could represent increased absorption or retention while decreasing urinary or faecal excretion appears to be the homeostatic mechanism by which the body retains greater amounts of Se [14]. However, our pair-fed dams have lower serum Se levels. In any case it is clear that ethanol *per se*, and not only malnutrition affects Se bioavailability in dams and therefore their Se deposits.

### 2.2. Pups' Weight at Birth and After Breastfeeding, and Pups' Se Homeostasis

Looking at Table 2, no detrimental effects were observed on body weight at birth, but in spite of a normal birth weight, alcohol (p < 0.01) and pair-fed (p < 0.05) treatment during lactation was responsible for a growth deficit which was more exacerbated in AO than in PO (p < 0.05). Ethanol but not isocaloric diet, decreases the number of pups per litter. Se in milk and the amount of milk consumed during the suckling period was drastically reduced in AO and PO groups (p < 0.001) and consequently their Se intake also decreased. This intake was even lower in AO than in PO (p < 0.001). There were no differences in the amount of Se excreted via faeces, but the PO excrete less Se via urine than the rest of the groups (p < 0.001), and more via hair than AO (p < 0.001). These changes in Se excretion alters serum Se levels, therefore AO had higher serum Se levels than CO (p < 0.01) and PO (p < 0.001), while PO levels lower than those of CO (p < 0.001) (Table 2).

The changes provoked in the dams’ Se bioavailability by ethanol, especially ethanol’s action over the mammary gland (where it decreases Se concentration), affect their progeny’s Se balance. Though AD has fewer pups per litter than CD, ethanol consumption does not alter their progeny weight at birth. However, at the end of the suckling period the body weight of pups whose mothers consumed less Se was lower, especially among those exposed to ethanol. This is provoked by a lower Se intake during the lactation period, itself caused by a decrease in Se milk content and a decrease in the amount of milk consumed during the breastfeeding process. Therefore the effect of Se malnutrition is more severe in pups than in their progenitors: ethanol and isocaloric dams consume 30% less Se than the control ones, but their progeny consumed 50%, nearly 60%, less than in the case of ethanol-exposed pups. Again, pair-fed pups excreted less Se via urine and more via hair than ethanol ones, and yet again they have lower Se serum levels. However, ethanol pups showed higher serum Se levels, even higher than control pups. It is known that in humans, hair Se concentration is higher in newborn babies than in their mothers by about 1.73-fold [15], in our control rats the same also occurs. However, in ethanol exposed rats the ratio increase to nearly 4.2-fold, and in pair-fed this increase is only 1-fold. We assumed that one of the mechanisms that changes Se bioavailability in ethanol pups, apart from the lowest Se intake, is the action of ethanol on urine and hair Se concentration. So despite the fact that some authors defend that hair and claw Se concentration are a reflection of dietary Se status [16,17], our results agree with Salbe *et al.* [18], who suggest that factors other than dietary Se intake affect hair and claw Se content and that these tissues should be used with caution for Se status assessment purposes.

### 2.3. Selenium Deposits in Different Tissues of Dams and Offspring (μg/g Dry Weight)

Tissue Se distribution in dams was altered by ethanol consumption (Table 3). Alcohol-receiving dams had significantly higher Se levels than the rest of the groups in heart, liver and spleen; and significantly lower Se levels than control ones in muscle (p < 0.01) and mammary glands (p < 0.05). PD have lower Se levels than control dams in muscle (P < 0.001), mammary gland (p < 0.05), lung (p < 0.05), kidney (p < 0.001) and heart (p < 0.001). Se distributions in offspring organs were lower in those exposed to malnutrition (AO and PO), with lower Se levels in heart, liver and kidney, in the last two tissues PO had significantly lower Se levels than AO (p < 0.001).

The Se levels in the organs of control dams were, in order of decreasing concentrations: kidney > spleen > heart, liver > mammary gland, muscle and lung. The lower Se retention provoked by alcohol consumption decreases Se levels in mammary gland and muscle. However, ethanol increases Se levels in spleen, heart and liver. It appears, therefore, that ethanol decreases Se levels in the tissues with lower Se deposits while sequestering Se to the spleen, heart and liver. According to a different bibliography [19–21], we thought that ethanol-exposed dams tended to maintain more Se in spleen, heart and liver at the expense of other tissues in order to improve their oxido-reduction balance, oxidation status and immunity, all of which are altered by ethanol consumption. Isocaloric lactating dams do not sequester Se to any of the studied organs, and they have lower Se levels than control ones in all of the tissues except the liver and spleen.

The order of decreased concentrations of Se deposits in the organs studied is different in dams and their offspring. In control pups the order was: kidney > liver > lung > spleen and heart, it seems that the Se requirements of tissues change with age, especially in the liver and lung. In these two organs Se deposits were higher in offspring than in their mothers; it seems that during development Se in liver and lung play an important role. Therefore the changes provoked by ethanol consumption in dams were different to those in their progeny. In suckling pups ethanol consumption decreases Se deposits in heart, liver and kidney, but it maintains the tissue order in decreasing Se concentration. However pair-fed offspring having lower Se levels in the same organs as ethanol treated ones: heart, liver and kidney, they showed lower Se levels in liver and kidney than ethanol pups. In this case the Se levels in their organs in order of decreasing concentrations were different to control and ethanol pups, being: lung > kidney > spleen > liver > heart. In these pups the levels of Se were especially compromised in liver and kidney. Curiously, in pups’ lung, Se levels were higher than in dams, nearly 1.3-fold. Excretion of Se can occur in the exhaled air [22], so lung, as hair and claw, are alternative tissues for the excretion of Se. It would appear that in pups, alternative routes (different to urine and faeces) participate more actively than during the adulthood in the elimination of Se.

### 2.4. Offspring Hepatic Glutathione Peroxidase Activity (A) and Serum Glutathione Peroxidase Activity (B)

**Figure 1.**
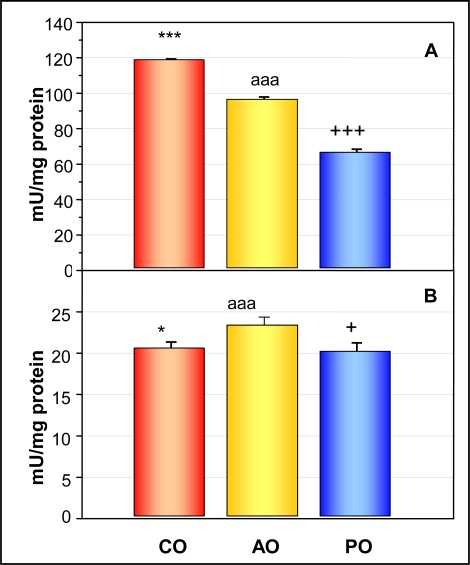
Offspring hepatic glutathione peroxidase (A) and serum glutathione peroxidase (B) activities (mU/mg protein). Signification: CO vs AO *p < 0.05, ***p < 0.001; AO vs PO ^aaa^p < 0.001; CO vs PO ^+^p < 0.05, ^+++^p < 0.001.

With respect to serum and hepatic GPx activity in offspring (Figure 1), alcohol-exposed pups showed higher serum GPx (p < 0.05) and lower hepatic GPx activity than control ones. PO pups had the lowest GPx activity in serum (PO vs AO p < 0.001 and PO vs CO p < 0.05) and in liver (p < 0.001).

Ethanol administered to pregnant mice disturbs embryogenesis by oxidative stress. This effect is more pronounced in the offspring of mice with a low antioxidative capacity [23]. As Se decreased oxidative stress through the GPx enzyme, mothers who had optimal levels during gestation and breastfeeding were able to prevent this effect. In this study we have found that AO have higher Se levels in serum and higher GPx activity than those found in the rest of the groups. It would appear that ethanol sequesters Se to the blood, perhaps in order to keep a high GPx activity in the serum, something that could play an important role in the first line of defence against ethanol-provoked oxidative stress. This hypothesis agrees with Payne and Southern [24], who suggested that Se stored in tissues could be utilized to maintain plasma GPx activity during periods of low Se intake. The action of ethanol on the GPx activity in liver was opposite to that which occurs in serum; it decreases hepatic Se levels and GPx activity, making it evident that oxidative stress is affected in pups whose mothers drank ethanol during gestation and lactation, and that this effect is related to Se levels. In pair-fed pups Se levels and GPx activity were drastically reduced in serum and liver. It is clear that ethanol *per se* alters both Se bioavailability and the oxidative balance in the offspring.

## 3. Experimental Section

**Animals:** Male and female Wistar rats (Centro de Producción y Experimentación Animal, Vicerrectorado de Investigación, Universidad de Sevilla), weighing 150–200 g, were randomised into three groups of dams: Control (CD), alcohol (AD), and pair-fed dams (PD) as a nutritional control of alcohol-associated malnutrition. CD: water and basic diet were given *ad libitum* during the entire experimental period. AD: ethanol and basic diet *ad libitum*. PD: water and an isocaloric diet with the diet of ethanol rats. These animals were used as parents. Male and female rats were mated to obtain the first generation offspring. Pregnant rats were housed individually in plastic cages. The day of parturition was designated as Day 1 of lactation, day 21 being the end of the lactation period. The offspring number was reduced to 8 per mother at parturition. The experiments were performed on dams and on their offspring at 21 days postpartum. The suckling pups were divided into three groups: CO: control, AO: pups exposed to ethanol during gestation and lactation, and PO: pair-fed offspring, born and suckled by their pair-fed dams. During the breastfeeding period, the pups had free access to the nipples. **Ethanol treatment:** Ethanol was administered in tap water at 20% v/v by a previously-described chronic method [25], during induction (4 weeks after 3 weeks naturalization), gestation (3 weeks) and lactation (3 weeks) periods. **Diets:** Diets were prepared according to The Council of the Institute of Laboratory Animal Resources (ILAR, 1979) which details the known nutrient requirements for most of the common laboratory animals (g/Kg of diet): Casein: 200; Granulated sucrose: 510; Cornstarch: 140; Fibre, cellulose: 50; Corn oil: 50; AIN-76 mineral mix: 35 (Albus; Córdoba, Spain) ; AIN-76 vitamin mix: 10 (Cecofar, Seville, Spain); Choline bitartrate: 2; dl-methionine: 3. Diet ingredients, including mineral and vitamin components, were mixed and homogenised in the laboratory in a double-cone blender (Rest Haan, Germany). The diet was offered to the animals in pellet form. The dams’ daily food consumption was determined by weighing the food offered and that remaining on a daily basis. Each measurement was taken at 9:00 AM to avoid changes due to circadian rhythms. **Se intake measurement:** Knowing the amount of food consumed and that the diet’s Se content 0.1ppm, we can calculate the amount of Se consumed. **Samples:** At the end of the experimental period, the rats were fasted for 12 hours and anesthetised with intraperitoneal 28% w/v urethane (0.5 mL/100 g of body weight). The abdomen was opened by a midline incision and different organs were removed, debrided of adipose and connective tissue in ice-cold saline, and weighed. Samples were immediately stored at −80 °C prior to determinations. Blood was collected by heart puncture and then centrifuged. Faeces and urine samples were collected using individual metabolic cages. **Milk samples:** In order to obtain the maximum amount of milk without modifying the physiological conditions of the subjects with anaesthetics on day 2 L of lactation, 3 h after removing the litters from their mothers, dams were sacrificed by decapitation and milk samples were immediately collected. Milk was obtained by gently massaging the area around each of the 12 mammary glands and then pressing upward from the base of the gland toward the nipple. **Milk consumption:** The amount of milk consumed was estimated by subtracting the weight of the pups obtained just prior to returning them to the dam from the weight at the end of 30 min of suckling. **Indexes:** The apparent Se absorption rate was calculated as [(I-F)/I] × 100 and the apparent Se balance as I-(F+U), where I = Se intake, F = Se faecal excretion and U = Se urinary excretion. **Selenium analysis:** Se levels were determined by graphite-furnace atomic absorption spectrometry. Equipment: We used a PerkinElmer AAnalyst™ 800 high-performance atomic absorption spectrometer with WinLab32 for AA software, equipped with a Transversely Heated Graphite Furnace (THGA) with longitudinal Zeeman-effect background corrector and AS-furnace autosampler (PerkinElmer, Ueberlingen, Germany). The source of radiation was an Se electrodeless discharge lamp (EDL). The instrumental operating conditions and the reagents are the same that we have used in the previous paper by Ojeda *et al.* [10] with slight modifications in the mineralization step: ramp time and temperature were different between tissues depending on their matrix content. Samples: serum samples were diluted fivefold in 0.2% v/v HNO_3_ and 0.2% Triton X-100 solutions and urine samples were diluted 1:2 v/v. After 72 h at 100 °C dry temperature, faeces, milk and different tissue samples were weighed and digested in a sand bath heater (OVAN) with nitric acid during 72 h, and added perchloric acid and chloridric acid (6N). After washing in Milli-Q water + acetone + acetone + acetone + Milli-Q water, claws and hair were treated in the same manner as the rest of the tissues. **Biochemical analysis.** Liver tissue samples were homogenized (2,500 rpm/min for 1 min, 1:10 w/v) (Pobel 245432, Spain) in a sucrose buffer (15 mM TRIS/HCl, pH 7.4, 250 mM sucrose, 1 mM EDTA, and 1 mM DTT) in an ice bath. The homogenate was centrifuged at 3,000 rpm for 10 min at 4 °C. The resulting supernatant was employed for the biochemical assay. Serum and hepatic samples were used to measure GPx activity. GPx activity was determined by spectrometry using Lawrence and Burk’s method [26] which based on GPx, catalyses the oxidation of glutathione by hydrogen peroxide. The protein content of the samples was determined by the method of Lowry *et al.* [27], using bovine serum albumin as the standard. **Statistical analyses:** The results are expressed as a mean ± SEM. The data were analysed using a statistical program (GraphPad InStat 3) by analysing the ANOVA parametric variance test followed by Tukey-Kramer tests. A p value of <0.05 was considered to be statistically significant.

## 4. Conclusions

We have concluded that ethanol decreased Se retention in Wistar rat dams, affecting their tissues’ Se deposits and those of their offspring. It also compromised the weight and the oxidation balance of their progeny. These effects of ethanol are caused by a reduction in Se intake and a direct alcohol generate-oxidation action.

## Figures and Tables

**Table 1.** Dams' weight gain and Se homeostasis: Se intake (μg/d) during gestation and lactation. Se levels in faeces, urine, claws, hair and serum. Se apparent absorption and balance.

	**CD (n:6)**	**AD (n:6)**	**PD (n:6)**

**Weight gain (g)**	78.3±7.1**	46.6±4.2	49.5±5.1^++^
**Se intake during gestation (**μ**g/d)**	1.89±0.21*	1.43±0.02	1.45±0.04^+^
**Se intake during lactation (**μ**g/d)**	3.12±0.33*	2.23±0.03	2.22±0.05^+^
**Se (**μ**g/d) in faeces**	0.26±0.02***	0.14±0.001	0.12±0.01^+++^
**Se (**μ**g/d) in urine**	0.18±0.01	0.23±0.02aaa	0.11±0.01^++^
**Se (**μ**g/l) in serum**	245 ± 15.2	259 ± 21.6aaa	150 ± 5.1^+++^
**Se (ng/g dry weight) in claws**	30 ± 3.6	30 ± 1.7aaa	66 ± 2.5^+++^
**Se (ng/g dry weight)in hair**	50 ± 0.5***	16 ± 1.0aaa	113 ± 8.0^+++^
**Apparent Se absorption rate (%)**	91.6±0.74	93.3±0.64	94.45±0.55^+^
**Apparent Se balance (**μ**g/d)**	2.67±0.07***	1.85±0.008^aa^	2.01±0.013^+++^

Signification: CD vs AD

* p < 0.05,

** p < 0.01,

*** p < 0.001; AD vs PD

^aa^ p < 0.01,

^aaa^ p < 0.001; CD vs PD

^+^p < 0.05,

^++^*p < 0.01,*

^+++^*p < 0.001.*

**Table 2.** Pups' weight at birth and at 21 days. Number of offspring per litter. Se in milk (ng/ml), milk intake in 30 min suckling (ml), Se intake during 30 min suckling (ng), Se levels in faeces, urine, hair and serum.

	**CO (n:8)**	**AO (n:8)**	**PO (n:8)**

**Weight at birth (g)**	5.53 ± 0.09	5.02 ± 0.18	5.14 ± 0.16
**Weight at 21 days (g)**	31.60 ± 1.05**	22.00 ± 1.67^a^	27.80 ± 1.15^+^
**Nº of offspring/ litter**	11.0±0.9*	7.8±0.6	9.0 ± 0.8
**Se (p.p.m.) in milk**	20 ± 1.2***	12.6 ± 0.6	13.7 ± 0.7^+++^
**Milk intake during 30 min suckling (mL)**	0.85 ±0.045***	0.54 ± 0.03	0.60 ± 0.04^+++^
**Se intake during 30 min suckling (ng)**	17.05 ± 0.1***	6.85 ± 0.03aaa	8.63 ± 0.04^+++^
**Se (ng/d) in faeces**	14 ± 0.1	18 ± 0.1	13 ± 0.1
**Se (ng/d) in urine**	15 ± 0.1	18 ± 0.1aaa	5 ± 0.05^+++^
**Se (**μ**g/L) in serum**	117 ± 3.4**	137 ± 6.9aaa	71.01±1.5^+++^
**Se (ng/g dry weight)in hair**	88 ± 0.7*	67 ± 0.5aaa	108 ± 0.7

Signification: CO vs AO

* p < 0.05,

** p < 0.01;

*** p < 0.001; AO vs PO

^a^ p < 0.05,

^aaa^ p < 0.001; CO vs PO

^+^*p < 0.05,*

^+++^*p < 0.001.*

**Table 3.** Selenium deposits in different tissues of dams and offspring (μg/g dry weight).

**Se levels (**μ**g/g dry weight)**	**CD (n: 6)**	**AD (n: 6)**	**PD (n: 6)**	**CO (n: 8)**	**AO (n: 8)**	**PO (n: 8)**

**Heart**	0.22±0.03**	0.27±0.05aaa	0.14±0.02^+++^	0.24 ± 0.01**	0.19 ± 0.01	0.13± 0.01^+++^
**Liver**	0.21±0.02*	0.28±0.02^a^	0.23±0.01	0.39±0.02***	0.30 ±0.01aaa	0.15±0.01^+++^
**Kidney**	0.66±0.04	0.63±0.04aaa	0.37±0.0^+++^	0.46±0.01***	0.37 ± 0.02aaa	0.26±0.01^+++^
**Lung**	0.14±0.008	0.13±0.003	0.12±0.007^+^	0.29±0.02	0.29±0.02	0.33±0.02
**Spleen**	0.28±0.01*	0.37±0.02^aa^	0.26±0.01	0.24±0.02	0.24±0.02	0.24±0.01
**Muscle**	0.15±0.01**	0.12±0.003	0.1±0.006^+++^			
**Mammary gland**	0.18±0.01**	0.13±0.005	0.14±0.005^+^			

Signification: C vs A

* p < 0.05,

** p < 0.01,

*** p < 0.001; A vs P

^a^ p < 0.05,

^aa^ p < 0.01,

^aaa^ p < 0.001; C vs P

^+^*p < 0.05,*

^+++^*p < 0.001.*
